# Supplementary figures and images for: The protective effect of DMI on hippocampus EEG, behavioral and biochemical parameters in hypoxia-induced seizure on neonatal period
Source: PLoS One. 2024 Nov 4;19(11):e0309240. doi: 10.1371/journal.pone.0309240 (PMC11534219; doi:10.1371/journal.pone.0309240)

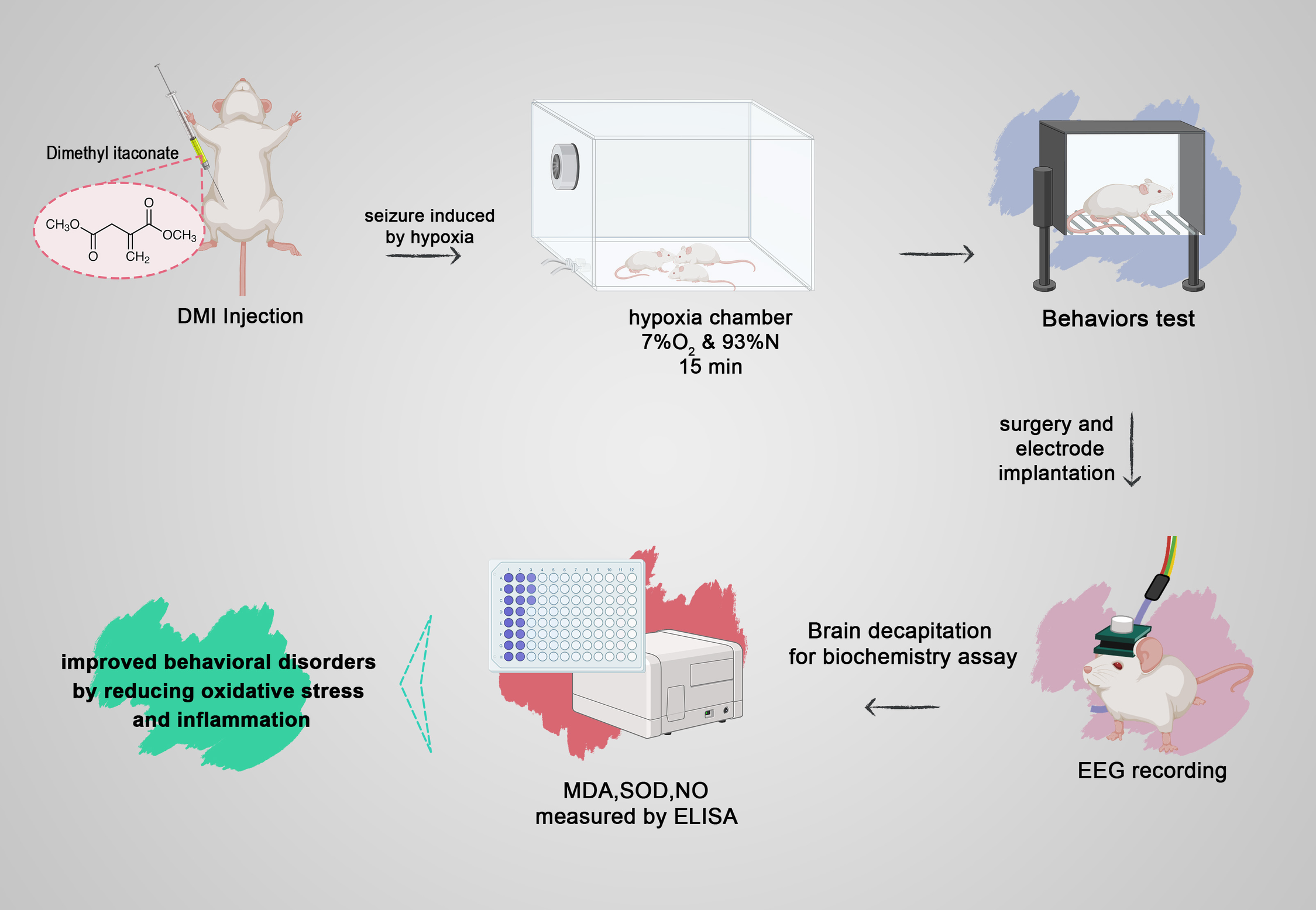

Supplement: S1 Graphical abstract — (TIF) [file pone.0309240.s002.tif]
