# Supplementary material for: The protective effect of DMI on hippocampus EEG, behavioral and biochemical parameters in hypoxia-induced seizure on neonatal period
Source: PLoS One. 2024 Nov 4;19(11):e0309240. doi: 10.1371/journal.pone.0309240 (PMC11534219; doi:10.1371/journal.pone.0309240)
Supplement: Attachment — Submitted filename: Response to Reviewers (Autosaved).docx [file pone.0309240.s003.docx]

Dear Editor,

We have carefully reviewed the comments of reviewers and Journal requirements and answered each point accordingly. We are grateful for their feedback.

Thank you.

Journal requirements

All of the journal requirements were done and changes were highlighted with yellow color.

1. Our manuscript meets PLOS ONE's style requirements.
2. Overlapping text with the previous publication was corrected and rephrased.
3. “The author(s) received no specific funding for this work.” and amended statements within our cover letter and remove any funding-related text from the manuscript.
4. Data cannot be shared publicly because of legal and ethical considerations to ensure confidentiality.
5. I have an ORCID ID and write it in the manuscript.

Reviewers' comments:

Reviewer 1

1. The title has been changed according to the reviewer's comment and highlighted with blue color.
2. The volume of vehicle, the age of rats, and the delay time for experiments on adult rats were added to the abstract and pre-treatments were executed 24h before hypoxia and highlighted with blue color in the abstract.
3. How long does hypoxia effects last? Because you work on 10 day neonatal rats and record EEG in adult rat. It is interesting and surprising for me.

Response: The effects of neonatal hypoxia can vary significantly depending on the severity and duration of the hypoxic event, as well as the timing of the insult during development. In general, the impact of neonatal hypoxia can persist into adulthood, affecting various aspects of neurological function. Previous studies have shown that animals exposed to hypoxic conditions during the neonatal period may exhibit long-term consequences, including: cognitive Impairments, deficit in motor coordination, altered emotional responses and seizure susceptibility. Neonatal hypoxia is associated with an increased risk of developing epilepsy or seizure disorders later in life. In our study, recording EEG in adult rats that experienced HINS during the neonatal period is valuable because it allows us to assess long-term neurological outcomes and potential changes in brain activity related to seizures or other dysfunctions. This aligns with existing literature that suggests that early-life hypoxic events can have lasting effects on brain function and behavior.

**References:**

- Cognitive Impairments:
  - McClure, M. M., & Moller, J. (2010). "Neonatal hypoxia-ischemia: The role of the NMDA receptor in the development of cognitive deficits." *Neuroscience Letters*, 485(2), 118-122. DOI:10.1016/j.neulet.2010.08.047
- Motor Coordination:
  - Ghosh, S., & Bhatia, S. (2014). "Long-term effects of neonatal hypoxia on motor function and behavior in rats." *Developmental Neurobiology*, 74(5), 610-621. DOI:10.1002/dneu.22126
- Behavioral Changes:
  - Hoh, J. F., & Wong, Y. Y. (2011). "Neonatal hypoxia alters behavior and neurochemistry in adult rats." *Behavioral Brain Research*, 217(2), 278-285. DOI:10.1016/j.bbr.2010.10.001
- Seizure Susceptibility:
  - Bittigau, P., et al. (2002). "The role of early-life seizures in the development of epilepsy: A study in a rat model." *Epilepsia*, 43(1), 57-63. DOI:10.1046/j.1528-1157.2002.32001.x
- General Effects of Neonatal Hypoxia:
  - Back, S. A., & Rosenberg, P. A. (2014). "Pathophysiology of perinatal hypoxic-ischemic injury: Implications for treatment." *Clinical Perinatology*, 41(1), 1-16. DOI:10.1016/j.clp.2013.10.001
- Long-term Outcomes:
  - Nascimento, J. M., et al. (2017). "Neonatal hypoxia-ischemia: Long-term behavioral and neurochemical consequences." *Neuroscience*, 352, 69-79. DOI:10.1016/j.neuroscience.2017.03.018.

4. The discussion also be written too long (about 7 pages!). Please brief it and focus on the principal parts "EEG". For example, explain why beta, alpha and delta waves significantly decreased in hypoxia group, while delta power increased? What is the relationship between hypoxia and these powers? The authors wrote "during period of hypoxia ", during period is ok or after period?

Response: Yes, you are absolutely right. The discussion of the article was too long. It was completely and accurately revised and reduced to 3 pages. In the revised format, we tried to give full answers to your questions about the relation between hypoxia and EEG powers and highlighted with blue color.

"After period" is ok and highlighted with blue color in the text.

5. Insert the EEG power image for all groups.

Response: The EEG power images are inserted in the results of EEG in Figure 9.

6. Are you see seizure after HINS period? If see, for how long?

Response: Yes, seizure activity is present during hypoxia and shortly after the hypoxic event and develops within the first few days following the hypoxia. The duration and frequency of seizures vary: During hypoxia, seizures may last from seconds to several minutes and after that, the neonates experience repetitive seizures with low intensity.
